# Radar detectors carried by Cape gannets reveal surprisingly few fishing vessel encounters

**DOI:** 10.1371/journal.pone.0210328

**Published:** 2019-02-06

**Authors:** David Grémillet, Julien Collet, Henri Weimerskirch, Nicolas Courbin, Peter G. Ryan, Lorien Pichegru

**Affiliations:** 1 Centre d’Ecologie Fonctionnelle et Evolutive, UMR 5175, CNRS—Université de Montpellier—Université Paul-Valéry Montpellier—EPHE, Montpellier, France; 2 Percy Fitzpatrick Institute of African Ornithology, NRF-DST Centre of Excellence at the University of Cape Town, Rondebosch, South Africa; 3 Centre d’Etudes Biologiques de Chizé, UMR 7372 CNRS–Université La Rochelle, 405 Route de Prissé la Charrière Villiers-en-Bois, France; 4 Institute for Coastal Marine Research and Department of Zoology, Nelson Mandela University, Port Elizabeth, South Africa; Phillip Island Nature Parks, AUSTRALIA

## Abstract

Fisheries compete with seabirds for vanishing marine resources, but also produce fishery waste consumed by seabirds. Marine birds may therefore avoid or seek fishing vessels, and have evolved complex, plastic behavioural responses to vessel presence. Understanding these responses is essential to the conservation of a globally declining seabird community. We studied Cape gannets (*Morus capensis*), which compete with fisheries for reduced sardine (*Sardinops sagax*) resources in the Benguela upwelling region off South Africa. Using bird-borne GPS trackers coupled with newly-developed ship-radar detectors we show that foraging gannets seldom attended fishing vessels. Rather, they switched from eating scarce sardines or energetically-poor fishery waste to targeting locally abundant saury (*Scomberesox saurus*). This pelagic fish is brought into the seascape by warm water influx, and is not commercially exploited by fisheries. Cape gannets thereby show dietary plasticity, allowing them to maintain adult body condition and chick growth rates. This diet switch is a strong indicator that Cape gannets forage in an ecologically perturbed marine environment.

## Introduction

Seabirds coexist with fisheries across the world’s oceans, and interact with them in a number of ways [1]. Seabirds have been guiding fishermen towards profitable harvesting grounds from ancient times. This practice persists to this day, even in industrial fisheries using radar technology to locate seabird aggregations over tuna shoals in tropical waters [2]. Further, fishing gear may catch or injure seabirds. This contributed to significant seabird population declines, especially in petrels and albatrosses exposed to long-line fisheries [3]. Mitigations measures have been relatively efficient with respect to hook-and-line fisheries [4], yet issues persist with other gear types and because mitigation measures on long-lines are rarely applied in international waters [5]. Also, 10% of fisheries catches are wasted at sea [6], and some of this biomass becomes additional food available to seabirds. Yet, fisheries also extract potential seabird food from the oceans, leading to competition for resources which are becoming limited because of global overfishing [7,8].

Seabirds have developed a range of responses to the presence of fishing vessels. Some species consistently show no reaction to the presence of any vessel (except to avoid them when resting at the water surface), especially those which feed on zooplankton (small petrels, prions and several alcids) and have nothing to gain from potential waste. In contrast, piscivorous seabirds are usually attracted by fishing vessels [9,10]. They are capable of discriminating gear types, and of showing contrasting responses to the presence of fishing vessels [11].

This is the case in Cape gannets (*Morus capensis*), which are endemic to the Benguela upwelling region off the west coast of southern Africa. In this marine ecosystem, previous work indicated that gannets avoided purse-seiners, with whom they compete for small pelagic fish, and actively sought trawlers, which generate fishery wastes upon which they can feed [12]. More specifically, Cape gannets are known to feed naturally by plunge-diving in coastal areas, primarily on sardines (*Sardinops sagax*). Following the fishery-induced collapse of this resource in the 1960s off South Africa, and in the 1970s off Namibia, gannets also fed on anchovies (*Engraulis encrasicolus*) and other prey [13]. In South Africa, small pelagic fish stocks (sardines and anchovies) partly recovered in the 1990s, but are still well below pre-exploitation levels [14].

Cape gannets presently compete with local purse-seine fisheries for greatly diminished sardine stocks [15]. In years with particularly low abundance of their natural prey, birds switch to feed on fishery wastes from demersal trawling vessels, primarily targeting hake (*Merlucius spp*.) [16]. This second fleet operates year-round within the foraging range of Cape gannets, performing 45–90 trawls per day, on average [17]. Fishing waste is easily available to gannets when dumped by vessels, yet its calorific value is only half that of sardines [18]. Adults feeding on this low-quality diet have poorer body condition, their chicks grow slower [16] and their reproductive output declines strongly [17].

Cape gannets therefore seem trapped in a system where they face a scarcity of their natural prey, and an abundance of ‘junk-food’. Their total population declined by 50–79% over 3 generations (approx. 50 years) and their conservation status has recently been downgraded to *Endangered*, considering this trend and the fact that they only occur at six breeding sites worldwide [19].

In order to better understand the foraging behaviour of Cape gannets in the context of limited food supplies, and to contribute to conservation schemes, we equipped them with recently-developed, miniaturized electronic tags. Those record the GPS position of birds travelling at-sea, but also regularly scan their surroundings to detect the presence of ship radar, as an indication of fishing vessel presence [20]. On the basis of previous work [12], we hypothesized that, faced with the scarcity of their natural prey, adult birds would approach vessels generating fishery waste.

## Methods

The study was conducted between the 24th of October and the 6^th^ of November 2017 on Malgas Island (33.05° S, 17.93° E) in the Western Cape, South Africa, which hosts some 19,000 breeding pairs of Cape gannets, this population having decreased from more than 50,000 pairs in the late 1990s [21]. All experiments were performed under permit from South African National Parks with respect to animal ethics (N° RYAP/AGR/001–2002/V1).

### Morphometrics and diet

Adult Cape gannets raising 2–4 week-old chicks were caught at the nest and handled in <10 min, whenever possible in the shade to avoid over-heating, and covering their heads to minimize stress. Adult body mass (±10 g) and wing length (±1 mm) were measured, and a body condition index calculated as body mass divided by wing length (g.mm^-1^), after Cohen and colleagues [16]. Some of the handled adults regurgitated food. These prey items were sampled opportunistically across the study period, and identified to species level. Prey item occurrence was used to calculate diet composition based on frequency of occurrence. The same sampling method had been used during the same time period and phase of the Cape gannet breeding cycle in previous years, allowing a comparison of the percentage of occurrence of the different prey items in their diet. This diet can change across the breeding period [22], and hence our data and conclusions concern the early stage of the breeding season only. Growth parameters of gannet chicks were determined during their linear growth phase, following Cohen and colleagues [16]: We calculated the difference in a chick’s mass between two consecutive measurements divided by the number of days between those two measurements. This growth rate was averaged when more than two measurements were taken per individual. Adult body condition indexes and chick growth rates were compared using GLMs with data collected through a standardized protocol (same colony, same time of year and same methodology in 2012–2015, including diet sampling; see [16]).

### Seabird tracking and vessel detection

Adult Cape gannets were fitted with one of two devices:

(1) GPS-logger (CatTrack1, Catnip Technologies, Hong-Kong, PRC, 70×32×15 mm, 30 g, 1.2% of bird body mass) attached to the lower back with waterproof Tesa tape, which recorded position and speed at 30-sec intervals.

(2) XGPS logger (Sextant Technology, New Zealand, 110×40×20 mm, 45 g, 1.7% of bird body mass) to record GPS locations every 1 min, but also to detect interactions between animals and vessels at sea by measuring Radar emission in the 9.41GHz X radar band which is used by marine radars. Radar signals are detected by an omnidirectional micro-strip antenna that integrates the signal over 30-sec every minute. Using the same devices, previous work [20] validated the method and showed that all vessels within 5 km of tracked wandering albatrosses (*Diomedea exulans*) were detected. Devices were recovered after 1–2 foraging trips lasting a few hours to 7 days. Bird handling and tracking using these procedures does not have a measurable impact on foraging behaviour [23]. We caught adult birds at-random from the colony. Cape gannets are monomorphic and share breeding duties at the nest. Hence there are no reasons to believe that one sex may have been preferentially caught, based on its appearance or accessibility.

**Fig 1:**
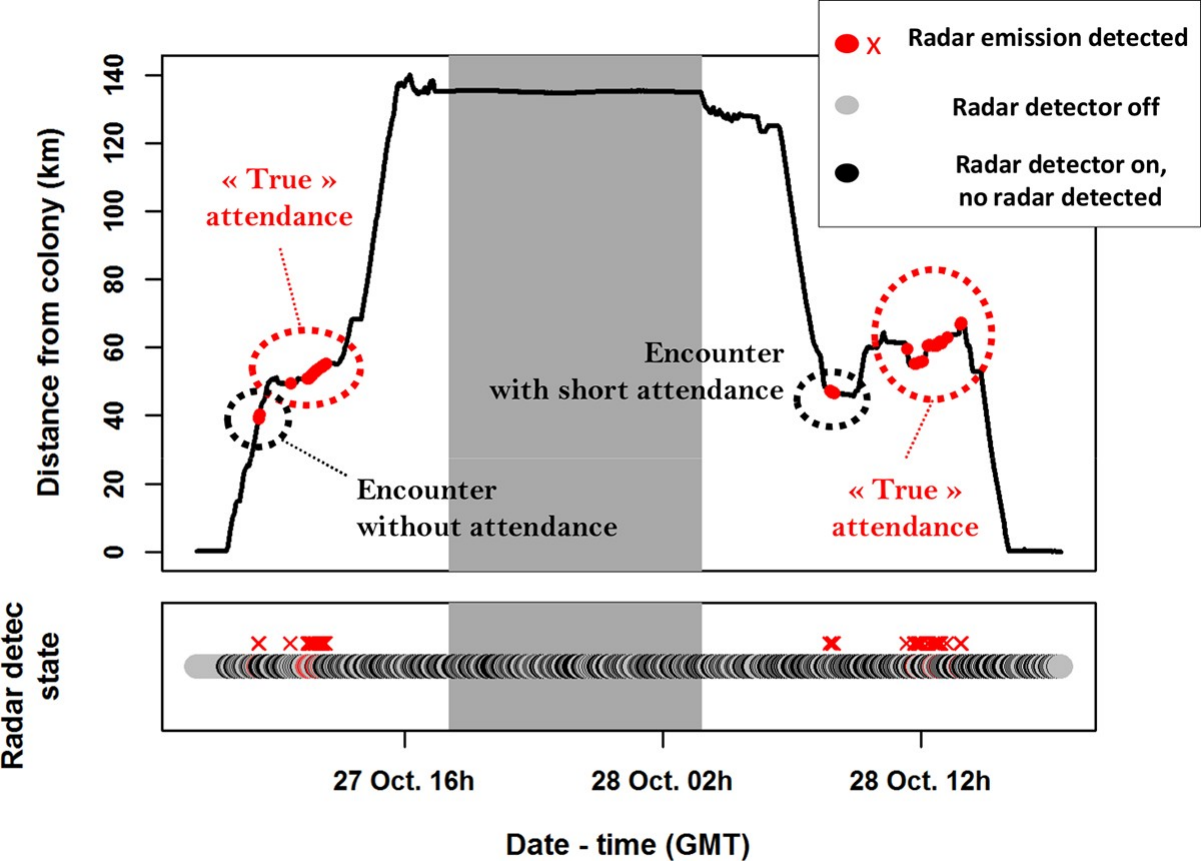
Example of radar detector data along a Cape gannet foraging trip recorded by a XGPS. Grey shaded areas correspond to night time. Four « encounters » separated from each other by more than 1h without radar detection were identified. Definitions for the different categories of encounters are provided in the Results section.

All recorded GPS tracks were resampled over 1-min intervals to harmonize the two data sets, and analysed following Grémillet and colleagues [23], who studied the same species. We thereby mapped seabird at-sea home-ranges (90% kernel contours) and calculated foraging trip duration, foraging path length, average foraging speed and maximum distance to the colony. From XGPS data, we examined the temporal distribution of successive radar detections, and grouped detections within 1 h of each other as the same vessel “encounter”. For each of these encounters, we extracted encounter duration (time between first and last boat radar detection without any gap > 1h, Fig 1), average bird speed, and the proportion of locations associated with a radar detection, between the first and last radar detection of the encounter. Observed co-variations in average speeds and encounter durations were used to visually categorize encounters in their likelihood to be “true” attendance when birds feed at vessels (see Results). Average values are reported ± SD.

### Fishing vessel home-ranges and Sea-Surface-Temperature (SST)

Data relative to the catch distribution of trawlers targeting hake and purse-seiners targeting anchovy and sardines were not available for our study period. We inferred the general distribution of operations for the two fishing fleets by mapping the catches as recorded in vessel log-books during 2008–2015 (10 x 10 nautical mile scale for purse-seiners; 20 x 20 nautical mile scale for trawlers). These data were made available by the branch: Fisheries Management of the Department of Agriculture, Forestry and Fisheries of the Republic of South Africa.

SST (°C) data were extracted from Aqua MODIS satellite imagery, with a 4 km-resolution grid (NASA Goddard Space Flight Center, Ocean Ecology Laboratory, Ocean Biology Processing Group; (2018): Aqua MODIS Sensor Ocean Color Data, NASA OB.DAAC.). Average SST was calculated for October-November each year across 2007–2017, within the core (90% UD) of Cape gannet at-sea home-range as determined through GPS-tracking during the 2007–2017 breeding seasons.

## Results

### Morphometrics and diet

**Fig 2:**
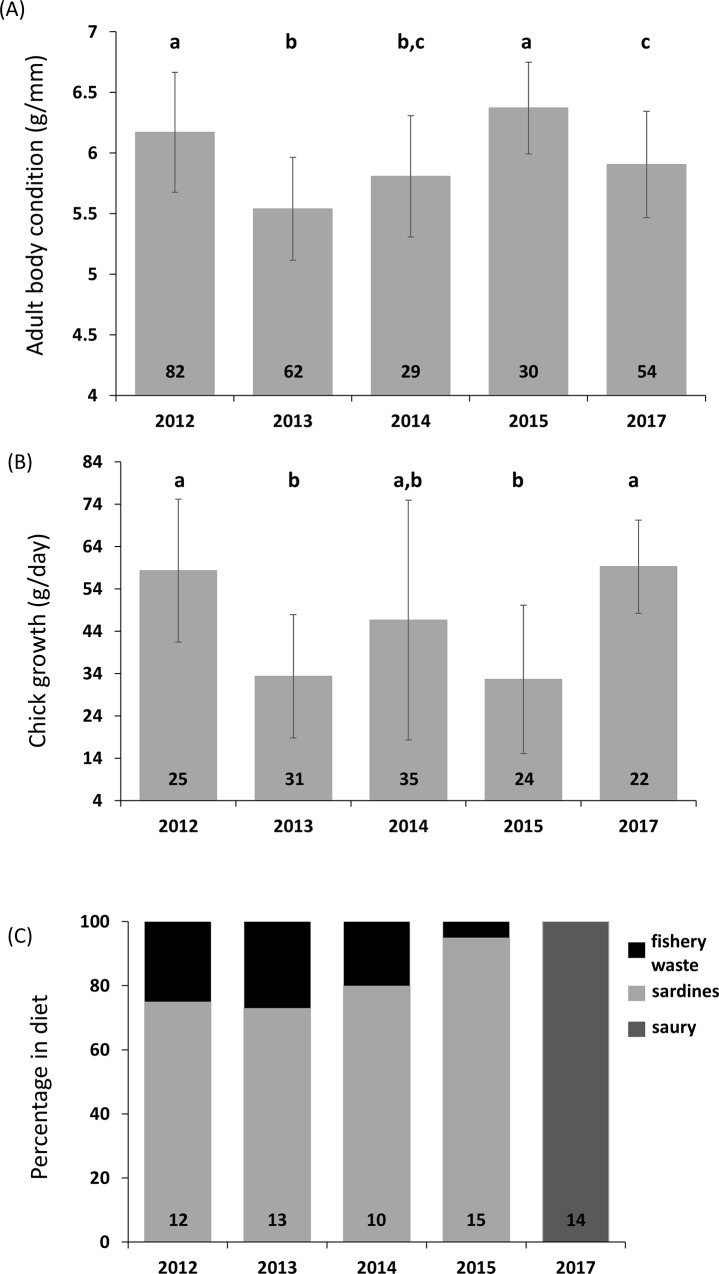
Cape gannet adult body condition index (bars: upper graph) and chick growth rates (bars: Middle graph), as well as Cape gannet diet (bars: Lower graph) for the 2012–2015 and 2017 breeding seasons on Malgas Island. Numbers at the bottom of histogram bars show sample sizes, and different letters above histogram bars denote statistically significant differences among years.

Adult body condition in 2017 was similar to indices measured during 2012–2015 (Fig 2). Chick growth rates were at the upper end of the range of values measured across 2012–2015 (Fig 2). All 14 food samples collected during the 2017 study period were Atlantic saury (*Scomberesox saurus*), a shoaling pelagic fish which primarily feeds on zooplankton and fish larvae, can reach a total length of 50 cm, and has a distinctively pointed rostrum. In previous years (2012–2015), gannet diet comprised 73–95% commercially exploited small pelagic fish, mainly sardines, the remainder of the diet being fishery discards (Fig 2).

### Seabird tracking and vessel detection

**Fig 3:**
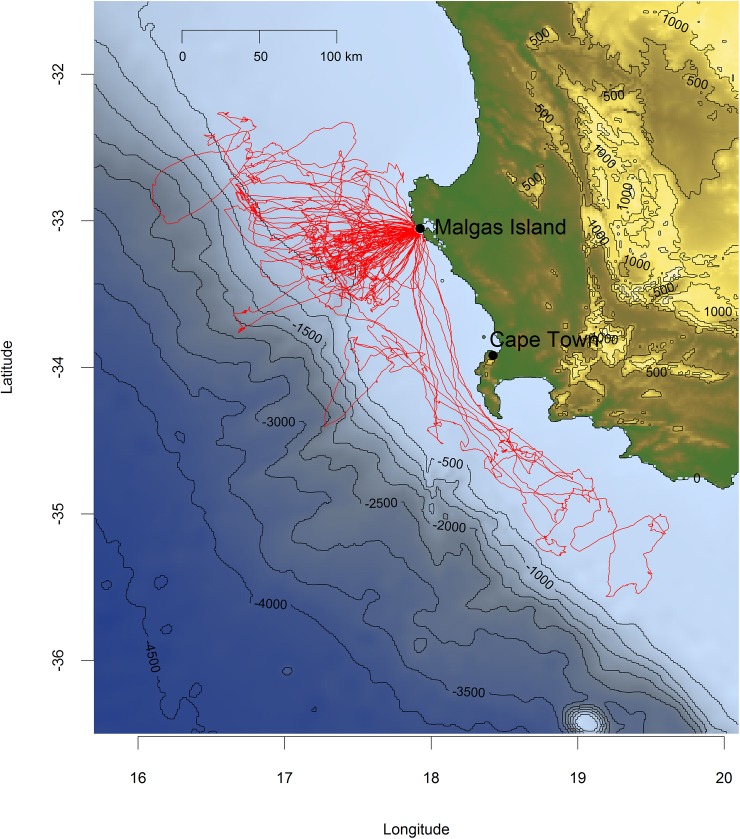
Foraging tracks of 50 Cape gannets provisioning for small chicks from the Malgas Island breeding colony in the southern Benguela upwelling region in 2017. Blue shades indicate bathymetry (R package marmap).

We tracked individual foraging trips of 50 Cape gannets lasting 20±24 h, covering an average total distance of 325±302 km, with an average maximum distance from the nest of 93±59 km. Birds foraged over the continental shelf in waters <1000 m deep, thereby remaining within the core of the Southern Benguela upwelling region (Fig 3). Their total home-range extended roughly 100 km west, and 200 km southeast of the breeding colony, with a strong focus on an area situated directly west of the breeding site (Fig 3).

Vessel encounter rates were determined for 20 birds equipped with XGPS (including one incomplete track due to battery failure). Thirteen gannets detected a radar signal. These birds travelled farther from the colony (average maximum range from the colony 121±52 km, n = 12 excluding the incomplete track) compared with trips when no radar was detected (68±27 km, n = 7, t = -2.917, df = 17, p = 0.01). For those 13 trips with recordings, radar signals were detected on 25±31 scans (median 10, max 98) conducted every minute. Overall, radar detections represented only 1.4% (range 0–3.8%) of foraging trip duration (but note that foraging trips also include non-feeding activities, such as commuting). Combining positive radar scans separated by less than 1 h without any detection, 28 radar “encounters” were detected.

**Fig 4:**
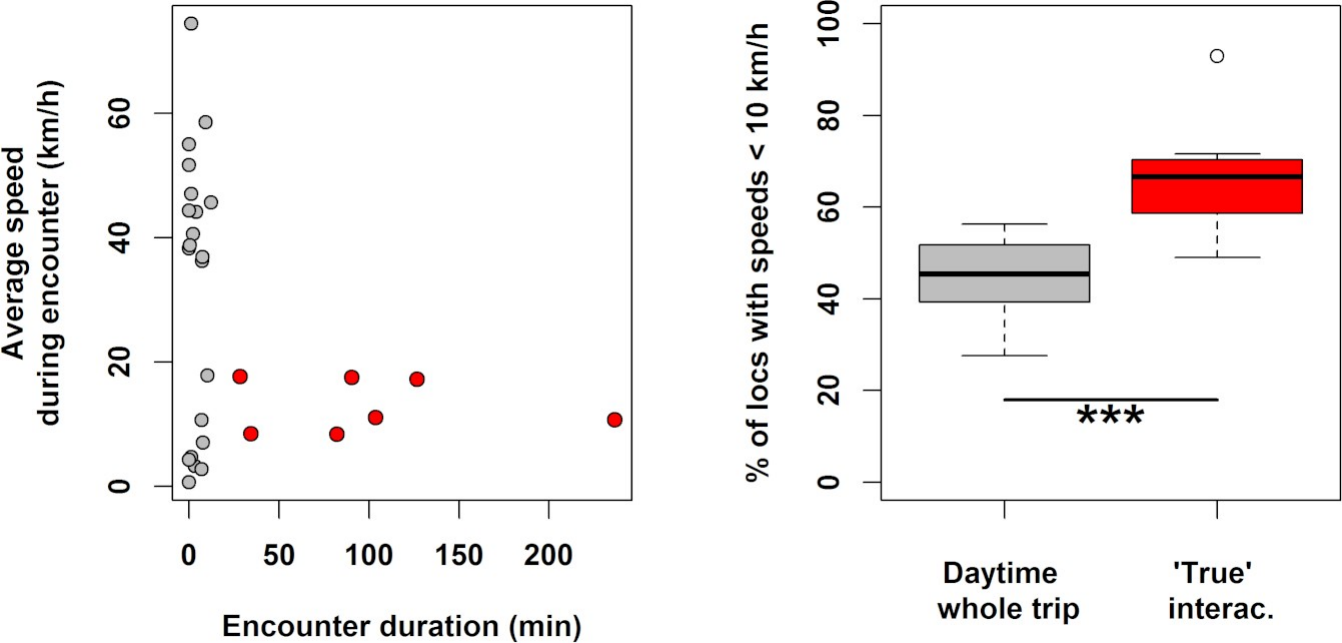
Vessel encounter characteristics. (Left): Average Cape gannet traveling speed and contact duration of 28 radar “encounters” by gannets (from 13 individuals), used for visual categorization; the seven encounters identified as true attendance (from 5 individuals) are highlighted in red. (Right): Proportions of speeds < 10km.h^-1^ during trips as a whole (during day time hours: left) or during the seven “true” attendance (right, red, ***: Wilcoxon test, p < 0.001).

**Fig 5:**
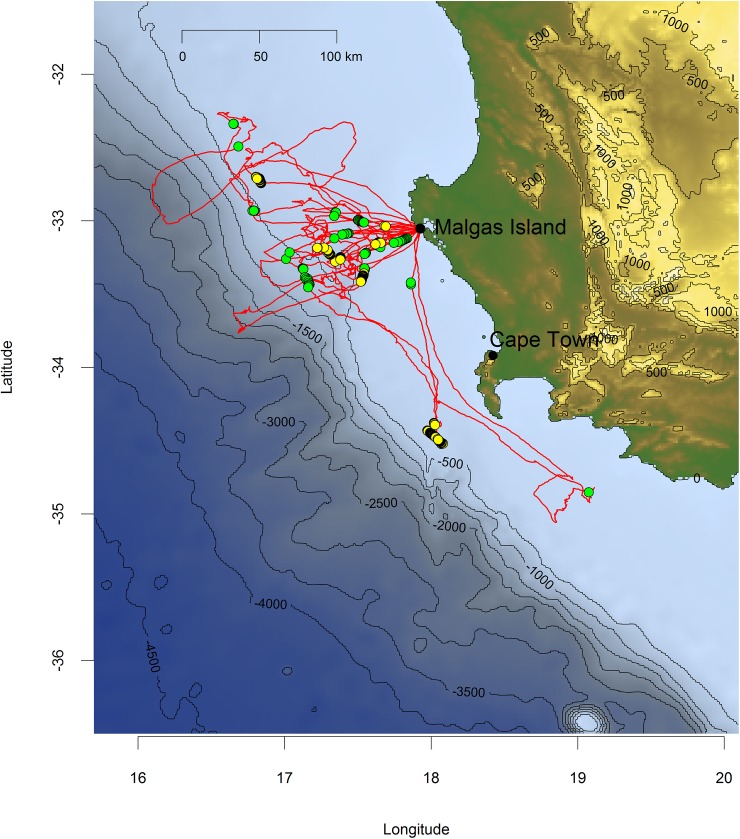
Foraging tracks of 13 Cape gannets during which vessel radar was detected. The seven detections with true bird-vessel attendance are indicated with yellow dots, whereas another 21 detections (green dots) featured short (<10 min) bird-vessel overlaps. Blue shades indicate bathymetry (R package marmap).

**Fig 6:**
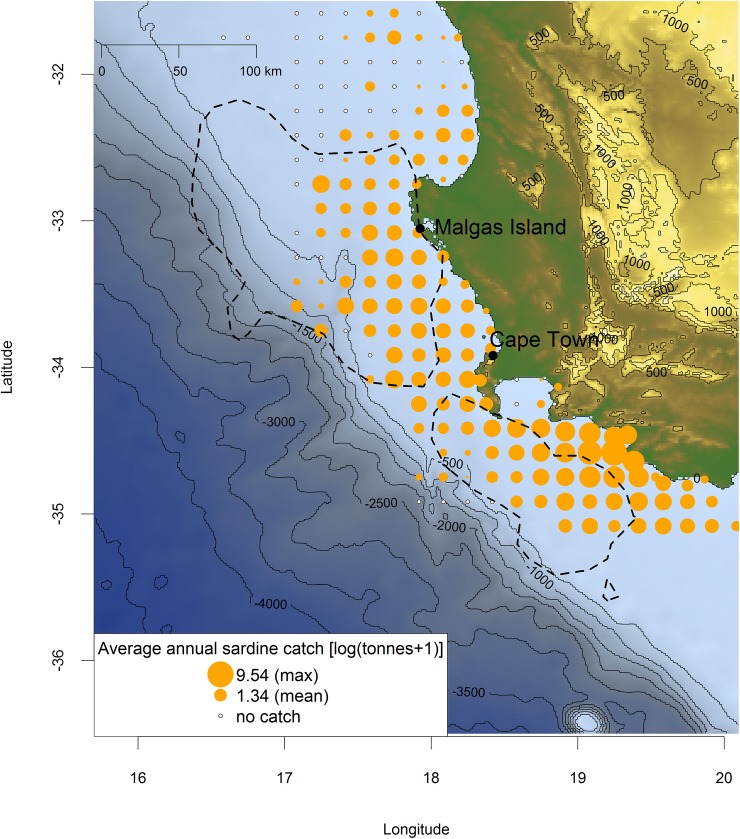
Overlap between the at-sea home-ranges of 50 GPS-tracked Cape gannets in Oct-Nov 2017 (90% kernel distributions, dotted lines) and average annual sardine catch by purse-seiners reported at a 10x10 nautical mile grid scale (log tonnes+1; orange dots) in 2008–2015. The size of orange dots increases with catch value from 0.20 to 9.54. White dots represent no catch in the fishing area. Blue shades indicate bathymetry (R package marmap).

**Fig 7:**
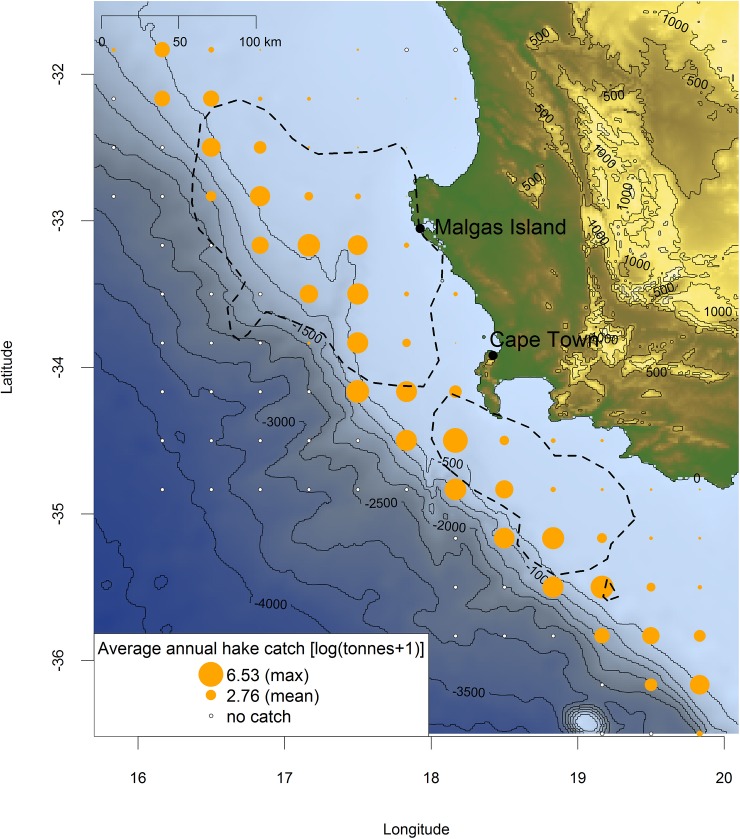
Overlap between the at-sea home-ranges of 50 GPS-tracked Cape gannets in Oct-Nov 2017 (90% kernel distributions, dotted lines) and average annual hake catch by trawlers in 2008–2015 (log tonnes+1; orange dots). The size of orange dots increases with catch value from 0.17 to 6.53. White dots represent no catch in the fishing area. Blue shades indicate bathymetry (R package marmap).

Thirteen were classified as encounters without attendance based on short detection periods by birds flying at commuting flight speeds (average speed 47±11 km.h^-1^, encounter duration 3.5±4.2 min, Fig 4). Eight encounters by six birds were categorized as short attendance, because the birds’ average speed decreased (6.5±5.5 km.h^-1^), consistent with foraging behaviour, but the interactions were of limited duration (<10 min). The remaining seven encounters by five birds were deemed to be “true” attendance behaviour to vessels based on their long duration (average 1.7±1.2 h, range 0.47–3.9 h, Fig 4) and low average travel speeds (13.0±4.3 km.h^-1^), which are typical speeds of fishing vessels (appreciably slower than merchant shipping, which is the other major category of shipping in these waters). The proportion of locations with an apparent speed < 10 km.h^-1^ (suggestive of sitting on water, as evidenced by night-time speeds: Fig 4) was higher during these attendance periods than on average during daytime (Wilcoxon test: W = 9, p<0.001, Fig 4). However only 29±14% of the duration of such encounters with “true” vessel attendance was spent within radar detection range. These attendance periods occurred within shelf waters, close to the 500 m isobaths, west and southwest of the colony, where trawling is fairly regular (Figs 5–7).

### Fishing vessel home-ranges and Sea-Surface-Temperatures (SST)

**Fig 8:**
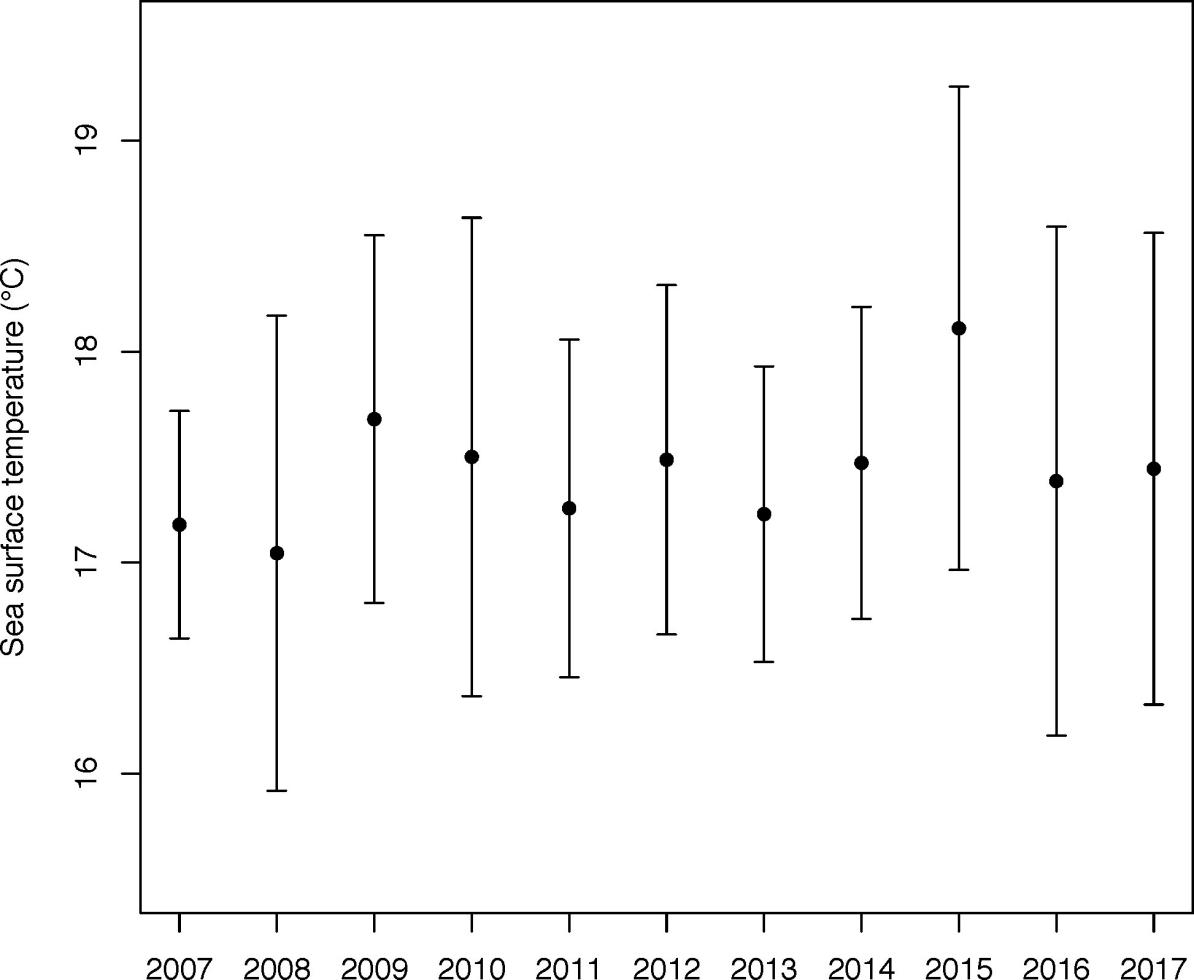
October sea surface temperature (°C; average with error bars showing SD) across 2008–2017 within the Malgas Cape gannet at-sea core home-range (90% kernel distribution), as determined via GPS-tracking of birds provisioning small chicks.

Data from fisheries log-books (2008–2015) showed that gannets visited the fishing areas targeted by both purse-seiners and trawlers (Fig 6). October-November SSTs (C°) recorded within Cape gannet core home ranges were consistently >16°C across 2007–2017 (Fig 8).

## Discussion

In contradiction with our hypothesis, foraging Cape gannets studied in the Benguela seldom encountered vessels in 2017, and even more rarely attended them for prolonged periods. This surprizing result can be explained through marked dietary plasticity, and has important implications for our understanding of seabird resilience and conservation in perturbed marine environments.

### Dietary switch

In the marine realm, some seabirds are known for their dietary plasticity [24], with recurrent dietary switches following fishery-induced perturbations [25–28]. This is precisely what we found in Cape gannets. During our study period, birds breeding on Malgas Island fed within their usual home-range off the west coast of South Africa (Fig 3; [23]), and their foraging parameters were comparable to those observed across the last decade [16]. Yet, surprisingly, they exclusively caught saury. Cape gannets normally favour sardines whenever available and switch to fishery waste (mainly hake) in the absence of their preferred prey [16]. Saury occurs irregularly in Cape gannet diet [13,22,29]. It favours warmer waters (18–22°C) than anchovy and sardine, which typically frequent upwelled waters (SST <16°C; [30]). The presence of saury close to the west coast of South Africa might therefore be a sign of reduced upwelling activity. However, the SST within the core home-range of foraging Cape gannets has been consistently >16°C during early chick-rearing (Oct-Nov) across the last decade (Fig 8), providing no support for abnormal conditions in 2017.

Because saury occur irregularly and in small schools [31], they are not exploited commercially in South Africa. Hence, Cape gannets targeting them are unlikely to forage in the vicinity of fishing vessels. The calorific value of saury (6.2 kJ.g^−1^) is intermediate between that of sardines (8.6 kJ.g^−1^) and hake (4.1 kJ.g^−1^; [32]), and diet calorific value strongly constrains gannet reproductive performance [17]. This potentially explains why gannets breeding on Malgas, facing a scarcity of sardines, switched to feeding on locally abundant saury, rather than fishery waste made available by hake trawlers. Dietary plasticity thus allows the gannets to maintain adult body condition and adequate chick growth rates (Fig 2; see [16,33] for further information on inter-annual trends). Saury has also been found in Cape gannet diet in Namibia [34], and is profitable prey for other Benguela seabirds, as indicated by a recent study [35] in greater crested terns (*Thalasseus bergii bergii*).

### Fishing vessel avoidance

Surprisingly few vessel detections occurred in gannets carrying XGPS recorders (Figs 4 and 5). This may seem puzzling considering the fact that Cape gannets share their home-range with two fishing fleets (trawlers and purse-seiners; Figs 6 and 7). As mentioned in the introduction, previous analyses combining gannet GPS-tracking with fishing vessel tracking via VMS, did show that birds avoided purse-seiners, with which they compete for small pelagic fish [12]. Yet, Tew-Kai and colleagues [12] also found that gannets actively sought, and followed trawlers, as a source of hake fishing waste when small pelagic fish were scarce. In the present study, only five of 20 birds carrying XGPS displayed prolonged attendance at vessels, and that for < 4% of their foraging time. These results are coherent with a seabird diet dominated by saury, which the birds could exploit without approaching vessels.

Cape gannets naturally balance their harvesting pressure across a range of potential prey, according to their availability and profitability [36,37]. Scarcity of particular prey types, including the most profitable ones, can arise from competition with other predators, including fisheries. Our findings occur in a marine ecosystem transformed by global changes, notably industrial fisheries and climate change [38]. In this context, prey switching is a strong indicator of reduced availability of sardines, which should be naturally abundant considering high primary and secondary productivity in the Benguela upwelling region, and used to be the food base of millions of Benguela seabirds [14]. Our results are coherent with reports on the 2017 state of South African hake trawl and small pelagic fisheries [39–41]. Indeed, those show that hake catches remained between ca. 130,000–140,000 tonnes across 2012–2016, and total allowable catch (TAC) was set to 140,125 tonnes for 2017 (despite decreasing hake recruitment). This strongly suggests sustained supply of fishery wastes to Cape gannets. On the contrary, a June 2017 sardine recruitment biomass survey found “the second lowest measured since May 2008 amounting to just over 23 000 tonnes and substantially lower than the long term average of ~143 000 tonnes”. Therefore, a sardine TAC of ca. 58,700 tonnes was recommended, but the effective 2017 TAC for sardines was set at ca. 86,700 tonnes. Overall, these figures point to diminishing sardine availability to Cape gannets, and to persistent competition with small pelagic purse-seine fisheries.

### Management implications

Seabirds and other top predators are likely to perform dietary switches, and this response conforms with optimal foraging theory [42]. Such plasticity linked to foraging sustainability thresholds explains why natural predators seldom overharvest a resource. By comparison, fisheries, as they are currently operated, are less flexible, because they are driven by the value of specific products (e.g. bluefin tuna [*Thunnus thynnus]*), by subsidies artificially inflating fleet sizes, harvesting range and effort, and by corruption [43].

Our work throws a new light on behavioural responses of Cape gannets confronted with a shifting prey base, and onto environmental management in the Benguela. Notably, seabird dietary plasticity complicates the parameterisation of ecosystem models, and environmental management. Indeed, models are often built using set functional relationships describing numerical responses of seabirds to the availability of specific prey [7]. Diet switches and foraging plasticity as demonstrated in our study distort these functional relationships [44]. For example, in Cape gannets, an ecosystem model could predict decreased breeding success as a consequence of sardine shortage. The use of saury as an alternative resource challenge this assumption.
